# Supplementary figures and images for: Registered Report: How does art impact pain and stress? Exposure to multimodal art (Music + Visual) and music alone enhances pain tolerance more than visual art, but neither art form impacts autonomic or endocrine markers
Source: PLoS One. 2026 May 5;21(5):e0334060. doi: 10.1371/journal.pone.0334060 (PMC13143110; doi:10.1371/journal.pone.0334060)

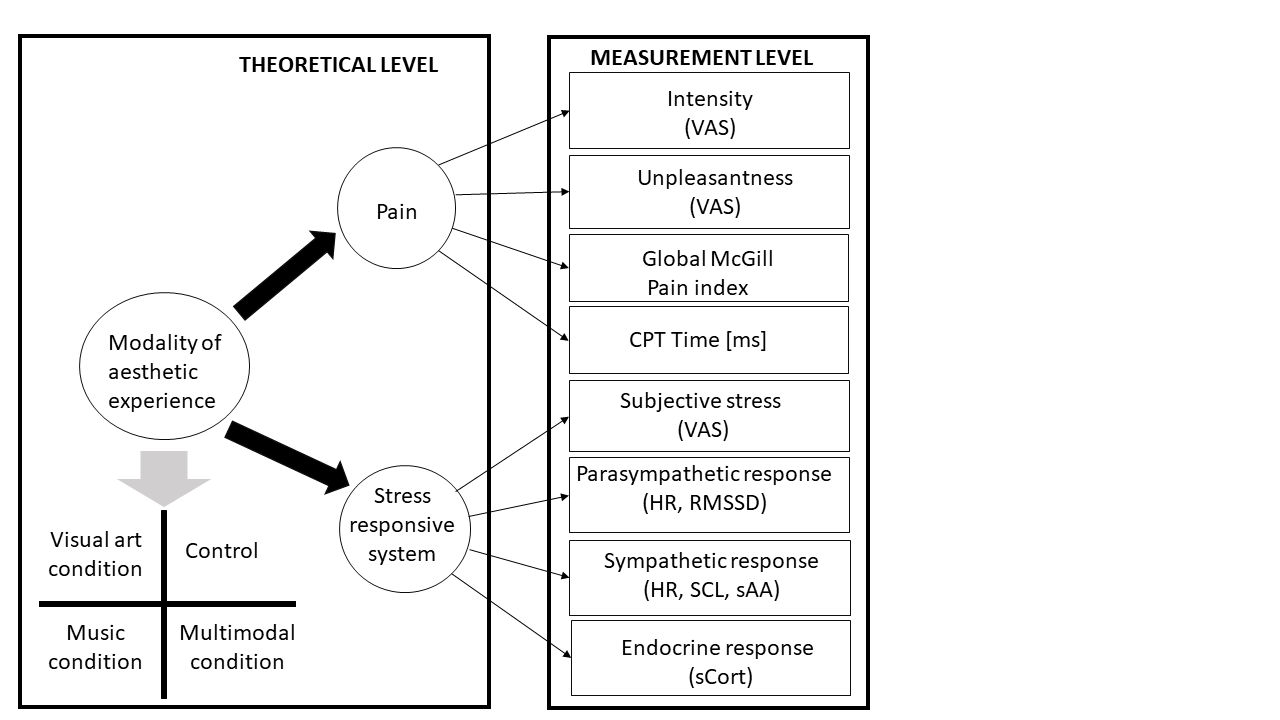

Supplement: S1 Fig — (TIF) [file pone.0334060.s001.tif]

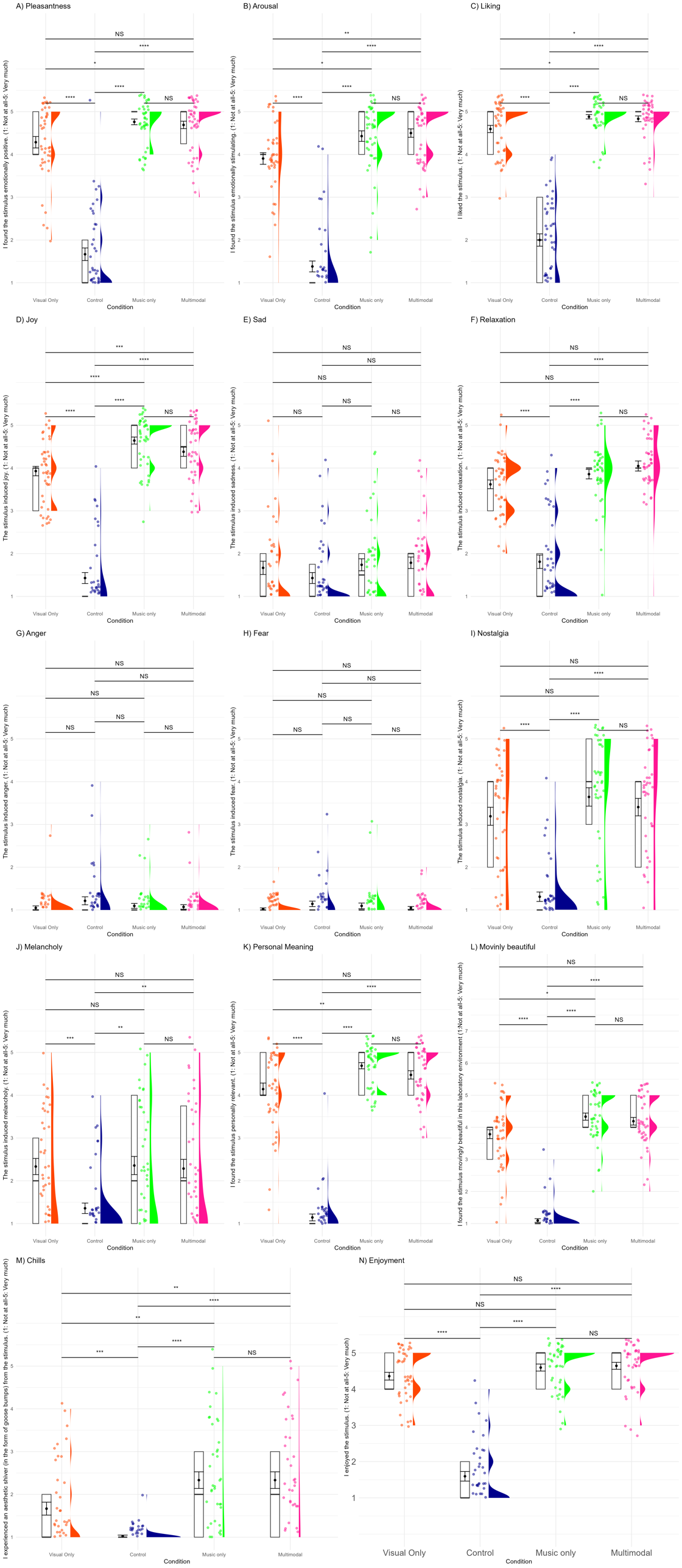

Supplement: S2 Fig — (TIF) [file pone.0334060.s002.tif]

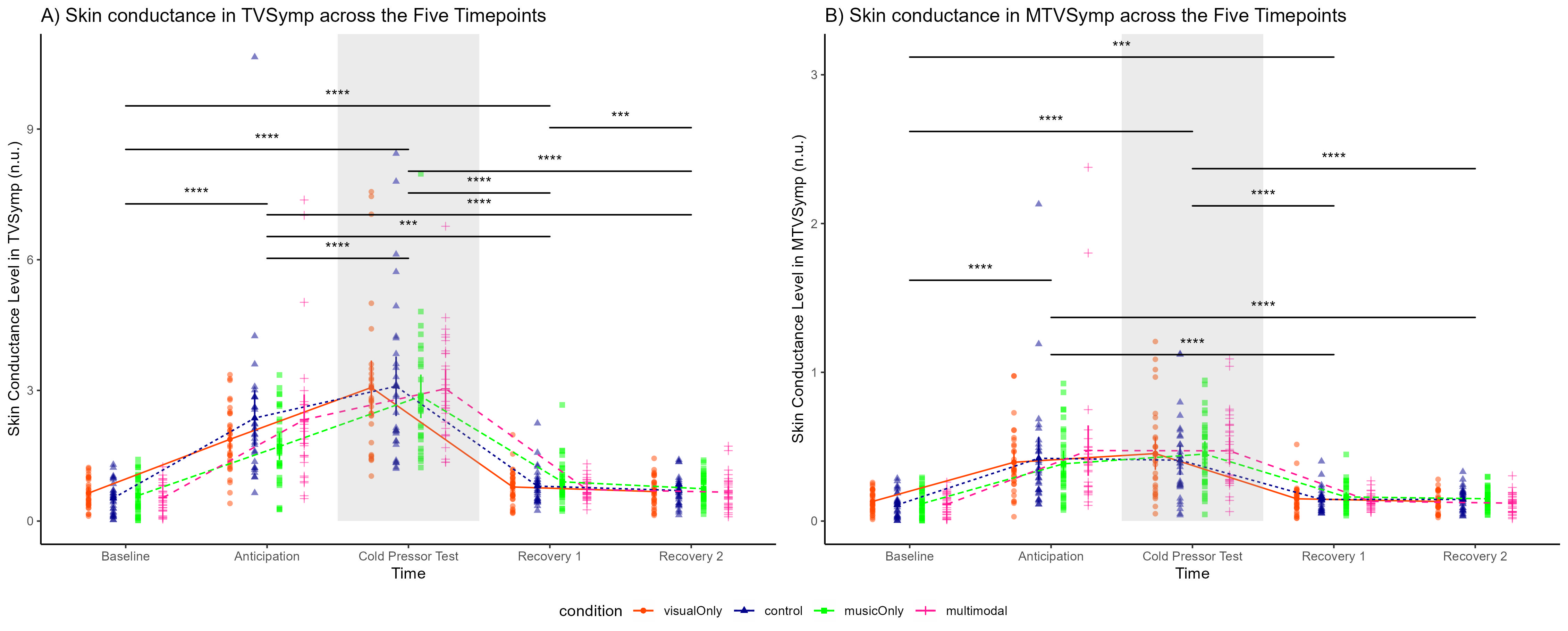

Supplement: S3 Fig — (JPG) [file pone.0334060.s003.jpg]
