# Supplementary material for: Registered Report: How does art impact pain and stress? Exposure to multimodal art (Music + Visual) and music alone enhances pain tolerance more than visual art, but neither art form impacts autonomic or endocrine markers
Source: PLoS One. 2026 May 5;21(5):e0334060. doi: 10.1371/journal.pone.0334060 (PMC13143110; doi:10.1371/journal.pone.0334060)
Supplement: S1 Table — (DOCX) [file pone.0334060.s004.docx]

**S1 Table. Summary of research questions, hypotheses, analysis plan and interpretation.**

| Question | Dependent Variable | Hypothesis | Analysis Plan | Interpretation given different outcomes |
| --- | --- | --- | --- | --- |
| 1.1. Does multimodal aesthetic experience (music + visual art) influence pain more than the single modalities?  1.2. Does musical aesthetic experience influence pain more than visual art? | Pain  (Tolerance, intensity, unpleasantness, global experience) | 1. 1. Multimodal aesthetic experience  1.1.1. increases pain tolerance,  1.1.2. decreases  a) pain intensity,  b) unpleasantness,  c) global McGill pain index  more than single modal aesthetic experience and control condition.  1.2. Music  1.2.1. increases pain tolerance,  1.2.2. decreases  a) pain intensity,  b) unpleasantness,  c) global McGill pain index  more than visual art and control condition. | One separate repeated measures ANOVA for each dependent variable will be calculated. For each dependent pain variable, one value will be investigated during the experimental task (tolerance in seconds, the other variables are reported retrospectively directly after the CPT).  *Condition* is included as a factor with four levels (music, visual art, multimodal, control).  If the repeated measures ANOVAs show significant effects, post-hoc tests using the Bonferroni correction are conducted to reveal between which conditions the significant difference has been found. | 1.1.1. If the multimodal condition shows higher pain tolerance (*p*≤ 0.05) to one or more other conditions, we will conclude that multimodal aesthetic experience increases pain tolerance.  Otherwise, we will conclude that it does not.  1.1.2. If the multimodal condition shows lower a) pain intensity, b) unpleasantness, c) global pain experience (*p*≤ 0.05) to one or more other conditions, we will conclude that multimodal aesthetic experience decreases that respective aspect (a, b, c) of pain experience. Otherwise, we will conclude that it does not influence one (or all included) aspects of pain experience.  1.2. Similarly, if music shows  1.2.1 more increased pain tolerance and  1.2.2. more decreased a) b) c) than visual art and the control condition, we conclude that music is more effective in influencing pain experience than visual art and the control condition. Otherwise, we will conclude that music is not more effective. |
| 2. 1. Does multimodal aesthetic experience (music + visual art) influence stress more than the single modalities?  2.2. Does musical aesthetic experience influence stress more than visual art? | Stress  (Subjective response; ECG; EDA; sAA; sCort) | 2.1.1. Multimodal aesthetic experience decreases stress. Specifically, we expect a decrease in:  a) *perceived subjective stress* (self-report: it refers to during the CPT attendance, which is retrospectively assessed directly after the CPT)  b) *sympathetic activity* (EDA measures in terms of SCL, sAA and HR)  c) *endocrine activity* (sCort)  2.1.2. Multimodal aesthetic experience increases:  d) *parasympathetic activity* (results in a decrease in HR and an increase in RMSSD)  during a pain test compared to single modal aesthetic experiences and control condition.  (In this case, all measures are compared to a Baseline value (see Fig 2- During the experiement)  2.2. Similarly, we expect that music  2.2.1. decreases stress more than visual art and a control condition, on a), b), c) aspects and  2.2.2. increases d) aspect.  (Those participants who attend the CPT shorter than 20 s are excluded from ECG and EDA analysis because quality of calculated physiological parameters cannot be ensured.) | One separate repeated measures ANOVA for each dependent variable will be calculated. *Condition* is included as a factor with four levels (music, visual art, multimodal, control).  If the repeated measures ANOVA shows significant effects, post-hoc tests using the Bonferroni correction are conducted to reveal between which conditions the significant difference has been found. | 2.1. If the multimodal condition  2.1.1. a) decreases perceived subjective stress, b) sympathetic activity, c) endocrine activity,  2.1.2. d) increases parasympathetic autonomic activity (*p*≤ 0.05) compared to one or more other conditions, we will conclude that multimodal aesthetic experience influences that respective aspect (a, b, c, or d) of stress experience.  Otherwise, we will conclude that it does not influence one (or all included) aspects of stress experience.  2.2. Similarly, if music  2.2.1. decreases stress on a), b), c) and  2.2.2. increases d) aspect more  than visual art and the control condition, we conclude that music influences that respective aspect (a, b, c, or d) of stress experience.  Otherwise, we will conclude that music is not more effective. |

ECG = electrocardiogram; HR = heart rate; HRV = heart rate variability; RMSSD = square root of the mean squared differences between successive heartbeat intervals; EDA = electrodermal activity; SCL = skin conductance level; sAA = salivary alpha-amylase; sCort = salivary cortisol.
