# Supplementary material for: Registered Report: How does art impact pain and stress? Exposure to multimodal art (Music + Visual) and music alone enhances pain tolerance more than visual art, but neither art form impacts autonomic or endocrine markers
Source: PLoS One. 2026 May 5;21(5):e0334060. doi: 10.1371/journal.pone.0334060 (PMC13143110; doi:10.1371/journal.pone.0334060)
Supplement: S2 Table — (DOCX) [file pone.0334060.s005.docx]

**S2 Table. Assessments in the whole course of the study.**

| Construct | Measure | Short name | Online survey previous to the study | Testing day 1 | Testing day 2 | Testing day 3 | Testing day 4 |
| --- | --- | --- | --- | --- | --- | --- | --- |
| *Primary variables* |  |  |  |  |  |  |  |
| Pain tolerance | Time [ms] |  |  | x | x | x | x |
| Pain intensity | Global McGill Pain Index  Visual Analogue Scale pain intensity | VAS pain intensity |  | x  x | x  x | x  x | x  x |
|  | Visual Analogue Scale momentary pain | VAS momentary pain |  | x | x | x | x |
| Pain affect | Visual Analogue Scale pain unpleasantness | VAS pain unpleasantness |  | x | x | x | x |
| *Secondary variables* |  |  |  |  |  |  |  |
| Acute subjective stress | Visual Analogue Scale stress intensity | VAS stress intensity |  | x | x | x | x |
|  | Visual Analogue Scale momentary stress | VAS momentary stress |  | x | x | x | x |
| ANS activity | ECG parameters |  |  | x | x | x | x |
|  | EDA parameters |  |  | x | x | x | x |
|  | sAA |  |  | x | x | x | x |
| Endocrine activity | sCort |  |  | x | x | x | x |
| Stimuli-related perceptions for the assessment of psychological mechanisms of aesthetic experience | Stimuli-related perceptions |  |  | x | x | x | x |
| Trait empathy | Questionnaire of Cognitive and Affective Empathy | QCAE | x |  |  |  |  |
| Trait absorption | Tellegen Absorption Scale | TAS | x |  |  |  |  |
| *Variables for description or checking of eligibility* |  |  |  |  |  |  |  |
| Demographic data | Questions on demographic data |  | x |  |  |  |  |
| Informed consent |  |  | x | x |  |  |  |
| Depression | Beck Depression Inventory | BDI-II | x |  |  |  |  |
| Menstrual cycle | Questions on menstrual cycle |  | x |  |  |  |  |
| Mental health | Patient health questionnaire | PHQ-D | x |  |  |  |  |
| Premenstrual syndrome | Premenstrual Syndrome Questionnaire | PMS questionnaire | x |  |  |  |  |
| Retrospective personal view on the study | Post-monitoring items (open-ended question regarding subjective experience) |  |  |  |  |  | x  (right after completing session 4) |

Short form of the McGill Pain Questionnaire (SF-MPQ-D) [1,2]; Beck Depression Inventory (BDI-II; [3]; Patient health questionnaire (PHQ-D;[4]; Premenstrual Syndrome Questionnaire (PMS questionnaire; [5]; German version of the Questionnaire of Cognitive and Affective Empathy (QCAE; [6]; Tellegen Absorption Scale (TAS; [7]; based on the original English version by [8].
