# Supplementary material for: Registered Report: How does art impact pain and stress? Exposure to multimodal art (Music + Visual) and music alone enhances pain tolerance more than visual art, but neither art form impacts autonomic or endocrine markers
Source: PLoS One. 2026 May 5;21(5):e0334060. doi: 10.1371/journal.pone.0334060 (PMC13143110; doi:10.1371/journal.pone.0334060)
Supplement: S3 Table — (DOCX) [file pone.0334060.s006.docx]

**S3 Table. Assessments during one testing day (one session).**

| Construct | Measure | Short name | During resting period | Directly after resting period | Directly before the CPT | During the CPT | Directly after the CPT | Recovery 1  (20 min after CPT start) | Recovery 2  (35 min after CPT start) |
| --- | --- | --- | --- | --- | --- | --- | --- | --- | --- |
| *Primary variables* |  |  | | | | | | | |
| Pain tolerance | Time [ms] |  |  |  |  | x |  |  |  |
| Pain intensity | Global McGill Pain Index  Visual Analogue Scale pain intensity | VAS pain intensity |  |  |  |  | x  x |  |  |
|  | Visual Analogue Scale momentary pain | VAS momentary pain |  | x | x |  | x | x | x |
| Pain affect | Visual Analogue Scale pain affect | VAS pain affect |  |  |  |  | x |  |  |
| *Secondary variables* |  |  | | | | | | | |
| Acute subjective stress | Visual Analogue Scale stress intensity | VAS stress |  |  |  |  | x |  |  |
|  | Visual Analogue Scale momentary stress | VAS momentary stress |  | x | x |  | x | x | x |
| ANS activity | ECG parameters |  | x | x | x | x | x | x | x |
|  | EDA parameters |  | x | x | x | x | x | x | x |
|  | sAA |  |  | x | x |  | x | x | x |
| Endocrine activity | sCort |  |  | x | x |  | x | x | x |
| Stimuli-related perceptions | Stimuli-related perceptions |  |  |  |  |  | x |  |  |

Short form of the McGill Pain Questionnaire (SF-MPQ-D) [1,2]; ECG = electrocardiogram; EDA = electrodermal activity; sAA = salivary alpha-amylase; sCort = salivary cortisol.
