# Supplementary material for: Registered Report: How does art impact pain and stress? Exposure to multimodal art (Music + Visual) and music alone enhances pain tolerance more than visual art, but neither art form impacts autonomic or endocrine markers
Source: PLoS One. 2026 May 5;21(5):e0334060. doi: 10.1371/journal.pone.0334060 (PMC13143110; doi:10.1371/journal.pone.0334060)
Supplement: S4 Table — (DOCX) [file pone.0334060.s007.docx]

**S4 Table. Momentary Pain VAS according to the Five Time Points**

| **Condition** | **I.**  **Baseline**  *M (SD)* | **II.**  **Anticipation**  *M (SD)* | **III.**  **After CPT**  *M(SD)* | **IV.**  **Recovery 1**  *M (SD)* | **V.**  **Recovery 2**  *M (SD)* |
| --- | --- | --- | --- | --- | --- |
| Visual | 2.24 (5.23) | 2.55 (5.15) | 46.26 (25.25) | 3.74 (7.79) | 3.10 (11.05) |
| Control | 1.83 (5.16) | 2.02 (5.51) | 52.29 (25.85) | 2.71 (5.59) | 1.26 (3.73) |
| Music | 1.55 (2.82) | 1.36 (2.60) | 52.00 (25.17) | 3.76 (8.41) | 1.14 (2.18) |
| Multimodal | 3.67 (9.50) | 3.05 (6.01) | 52.02 (22.16) | 3.76 (9.50) | 2.16 (6.08) |
| All | 2.32 (6.16) | 2.24 (4.99) | 50.64 (24.56) | 3.49 (7.89) | 1.92 (6.65) |

*Note: VAS: Visual Analogue Scale., CPT: Cold Pressor Test.*
