# Supplementary material for: Registered Report: How does art impact pain and stress? Exposure to multimodal art (Music + Visual) and music alone enhances pain tolerance more than visual art, but neither art form impacts autonomic or endocrine markers
Source: PLoS One. 2026 May 5;21(5):e0334060. doi: 10.1371/journal.pone.0334060 (PMC13143110; doi:10.1371/journal.pone.0334060)
Supplement: S5 Table — (DOCX) [file pone.0334060.s008.docx]

**S5 Table. Momentary Stress VAS according to the Five Time Points**

| **Condition** | **I.**  **Baseline**  *M (SD)* | **II.**  **Anticipation**  *M (SD)* | **III.**  **After CPT**  *M(SD)* | **IV.**  **Recovery 1**  *M (SD)* | **V.**  **Recovery 2**  *M (SD)* |
| --- | --- | --- | --- | --- | --- |
| Visual | 11.38 (15.89) | 9.71 (14.39) | 26.43 (20.82) | 8.26 (12.18) | 6.86 (14.07) |
| Control | 9.17 (15.64) | 11.95 (17.25) | 30.90 (22.01) | 8.38 (14.81) | 7.31 (12.72) |
| Music | 11.45 (20.22) | 12.64 (21.51) | 28.86 (22.62) | 9.55 (17.10) | 8.40 (17.29) |
| Multimodal | 12.55 (20.07) | 12.74 (20.51) | 25.07 (20.21) | 10.60 (16.69) | 7.60 (14.08) |
| All | 11.14 (17.97) | 11.76 (18.50) | 27.82 (21.36) | 9.20 (15.21) | 7.54 (14.52) |

*Note: VAS: Visual Analogue Scale., CPT: Cold Pressor Test.*
