# Supplementary material for: Registered Report: How does art impact pain and stress? Exposure to multimodal art (Music + Visual) and music alone enhances pain tolerance more than visual art, but neither art form impacts autonomic or endocrine markers
Source: PLoS One. 2026 May 5;21(5):e0334060. doi: 10.1371/journal.pone.0334060 (PMC13143110; doi:10.1371/journal.pone.0334060)
Supplement: S6 Table — (DOCX) [file pone.0334060.s009.docx]

**S6 Table. Heart Rate Variability in Square Root of the Mean Squared Differences of Successive** **Heartbeat Intervals (RMSSD) according to the Five Time Points**

| **Condition** | **I.**  **Baseline**  *M (SD)* | **II.**  **Anticipation**  *M (SD)* | **III.**  **During CPT**  *M (SD)* | **IV.**  **Recovery 1**  *M (SD)* | **V.**  **Recovery 2**  *M (SD)* |
| --- | --- | --- | --- | --- | --- |
| Visual | 63.68 (29.83) | 77.61 (42.20) | 69.38 (34.41) | 82.24 (36.55) | 72.85 (32.60) |
| Control | 63.38 (35.20) | 83.42 (48.06) | 62.20 (38.70) | 82.35 (36.63) | 69.66 (34.95) |
| Music | 65.37 (34.55) | 76.21 (43.78) | 51.33 (30.38) | 84.30 (39.65) | 68.84 (28.83) |
| Multimodal | 65.65 (36.85) | 82.99 (45.69) | 65.77 (37.66) | 79.57 (34.60) | 70.16 (28.76) |
| All | 64.52 (33.90) | 80.13 (44.61) | 62.51 (35.89) | 82.07 (36.48) | 70.41 (31.18) |

*Note: CPT: Cold Pressor Test.*
