# Supplementary material for: Registered Report: How does art impact pain and stress? Exposure to multimodal art (Music + Visual) and music alone enhances pain tolerance more than visual art, but neither art form impacts autonomic or endocrine markers
Source: PLoS One. 2026 May 5;21(5):e0334060. doi: 10.1371/journal.pone.0334060 (PMC13143110; doi:10.1371/journal.pone.0334060)
Supplement: S7 Table — (DOCX) [file pone.0334060.s010.docx]

**S7 Table. Heart Rate in Beat Per Minute (BPM) according to the Five Time Points**

| **Condition** | **I.**  **Baseline**  *M (SD)* | **II.**  **Anticipation**  *M (SD)* | **III.**  **During CPT**  *M (SD)* | **IV.**  **Recovery 1**  *M (SD)* | **V.**  **Recovery 2**  *M (SD)* |
| --- | --- | --- | --- | --- | --- |
| Visual | 72.78 (12.44) | 76.75 (11.69) | 77.46 (12.35) | 71.37 (11.32) | 69.60 (11.59) |
| Control | 74.69 (10.83) | 80.81 (12.34) | 80.18 (13.06) | 72.52 (11.18) | 72.08 (11.73) |
| Music | 71.20 (11.31) | 76.47 (13.39) | 79.68 (13.70) | 69.78 (10.43) | 67.53 (10.76) |
| Multimodal | 73.63 (11.24) | 77.67 (14.09) | 82.35 (11.09) | 72.40 (9.63) | 72.45 (9.73) |
| All | 73.13 (11.42) | 77.95 (12.89) | 79.98 (12.53) | 71.54 (10.61) | 70.43 (11.07) |

*Note: CPT: Cold Pressor Test.*
