# Supplementary material for: Registered Report: How does art impact pain and stress? Exposure to multimodal art (Music + Visual) and music alone enhances pain tolerance more than visual art, but neither art form impacts autonomic or endocrine markers
Source: PLoS One. 2026 May 5;21(5):e0334060. doi: 10.1371/journal.pone.0334060 (PMC13143110; doi:10.1371/journal.pone.0334060)
Supplement: S8 Table — (DOCX) [file pone.0334060.s011.docx]

**S8 Table. Skin Conductance Level (SCL) in μS according to the Five Time Points**

| **Condition** | **I.**  **Baseline**  *M (SD)* | **II.**  **Anticipation**  *M (SD)* | **III.**  **After CPT**  *M (SD)* | **IV.**  **Recovery 1**  *M (SD)* | **V.**  **Recovery 2**  *M (SD)* |
| --- | --- | --- | --- | --- | --- |
| Visual | 2.20 (1.22) | 3.20 (1.77) | 3.39 (1.88) | 2.93 (1.74) | 2.85 (1.79) |
| Control | 2.09 (1.29) | 3.28 (1.84) | 3.49 (1.96) | 2.92 (1.62) | 2.81 (1.58) |
| Music | 2.34 (1.56) | 3.32 (1.90) | 3.59 (2.08) | 3.24 (1.94) | 3.19 (1.95) |
| Multimodal | 2.01 (1.22) | 3.01 (1.65) | 3.24 (1.80) | 2.80 (1.59) | 2.73 (1.63) |
| All | 2.16 (1.32) | 3.20 (1.78) | 3.42 (1.92) | 2.97 (1.72) | 2.90 (1.74) |

*Note: CPT: Cold Pressor Test.*
