# Supplementary material for: Registered Report: How does art impact pain and stress? Exposure to multimodal art (Music + Visual) and music alone enhances pain tolerance more than visual art, but neither art form impacts autonomic or endocrine markers
Source: PLoS One. 2026 May 5;21(5):e0334060. doi: 10.1371/journal.pone.0334060 (PMC13143110; doi:10.1371/journal.pone.0334060)
Supplement: S9 Table — (DOCX) [file pone.0334060.s012.docx]

**S9 Table. Salivary alpha-amylase (sAA) [U/ml] according to the Five Time Points**

| **Condition** | **I.**  **Baseline**  *M (SD)* | **II.**  **Anticipation**  *M (SD)* | **III.**  **After CPT**  *M (SD)* | **IV.**  **Recovery 1**  *M (SD)* | **V.**  **Recovery 2**  *M (SD)* |
| --- | --- | --- | --- | --- | --- |
| Visual | 82.50 (72.48) | 84.22 (57.39) | 99.65 (75.00) | 80.28 (62.85) | 81.85 (68.72) |
| Control | 76.68 (54.54) | 90.43 (72.26) | 94.80 (81.94) | 73.70 (52.61) | 72.81 (50.86) |
| Music | 90.71 (82.37) | 93.24 (77.10) | 113.35(102.30) | 93.68 (67.51) | 87.36 (64.68) |
| Multimodal | 86.32 (73.18) | 93.56 (78.40) | 96.53 (72.12) | 88.64 (77.03) | 105.77(93.83) |
| All | 83.97 (70.71) | 90.38 (71.25) | 101.16 (83.36) | 84.05 (65.42) | 89.48 (78.89) |

*Note: CPT: Cold Pressor Test; VAS: Visual Analogue Scale.*
