# Supplementary material for: Registered Report: How does art impact pain and stress? Exposure to multimodal art (Music + Visual) and music alone enhances pain tolerance more than visual art, but neither art form impacts autonomic or endocrine markers
Source: PLoS One. 2026 May 5;21(5):e0334060. doi: 10.1371/journal.pone.0334060 (PMC13143110; doi:10.1371/journal.pone.0334060)
Supplement: S10 Table — (DOCX) [file pone.0334060.s013.docx]

**S10 Table. Salivary cortisol (sCort) [nmol/l] according to the Five Time Points**

| **Condition** | **I.**  **Baseline**  *M (SD)* | **II.**  **Anticipation**  *M (SD)* | **III.**  **After CPT**  *M(SD)* | **IV.**  **Recovery 1**  *M (SD)* | **V.**  **Recovery 2**  *M (SD)* |
| --- | --- | --- | --- | --- | --- |
| Visual | 4.02 (2.46) | 4.11 (2.48) | 3.81 (2.36) | 4.76 (3.34) | 3.91 (2.83) |
| Control | 4.08 (2.58) | 3.96 (2.64) | 3.93 (2.50) | 4.46 (3.37) | 4.26 (3.54) |
| Music | 4.25 (3.05) | 4.24 (2.75) | 4.12 (2.52) | 4.98 (2.70) | 4.55 (3.01) |
| Multimodal | 4.17 (2.34) | 4.19 (2.21) | 4.04 (2.19) | 5.18 (2.72) | 4.72 (3.11) |
| All | 4.13 (2.60) | 4.13 (2.51) | 3.98 (2.38) | 4.84 (3.03) | 4.36 (3.12) |

*Note: CPT: Cold Pressor Test; VAS: Visual Analogue Scale.*
