# Supplementary material for: Registered Report: How does art impact pain and stress? Exposure to multimodal art (Music + Visual) and music alone enhances pain tolerance more than visual art, but neither art form impacts autonomic or endocrine markers
Source: PLoS One. 2026 May 5;21(5):e0334060. doi: 10.1371/journal.pone.0334060 (PMC13143110; doi:10.1371/journal.pone.0334060)
Supplement: S11 Table — (DOCX) [file pone.0334060.s014.docx]

**S11 Table. Descriptive Data of the Mechanisms of Aesthetic Experience on Pain and Stress as well as Features of the Art**

|  | Condition | | | |
| --- | --- | --- | --- | --- |
| Measures | Visual  M (SD) | Control  M (SD) | Music  M (SD) | Multimodal  M (SD) |
| Distraction | 3.40 (0.94) | 1.64 (0.88) | 4.36 (0.62) | 4.38 (0.82) |
| Mind wandering | 3.67 (1.22) | 1.64 (0.98) | 4.24 (0.82) | 4.19 (0.99) |
| Art Pleasantness | 4.28 (0.86) | 1.67 (0.95) | 4.76 (0.43) | 4.69 (0.56) |
| Art Emotional Arousal | 3.90 (0.88) | 1.38 (0.82) | 4.43 (0.80) | 4.50 (0.63) |
| Art Liking | 4.60 (0.54) | 2.00 (0.94) | 4.88 (0.33) | 4.83 (0.44) |
| Art Joy | 3.93 (0.71) | 1.43 (0.83) | 4.64 (0.53) | 4.38 (0.70) |
| Art Sadness | 1.67 (1.00) | 1.43 (0.83) | 1.74 (0.91) | 1.79 (0.87) |
| Art Relaxation | 3.62 (0.66) | 1.81 (1.02) | 3.86 (0.72) | 4.05 (0.76) |
| Art Anger | 1.05 (0.31) | 1.21 (0.61) | 1.10 (0.37) | 1.07 (0.34) |
| Art Fear | 1.02 (0.15) | 1.14 (0.42) | 1.10 (0.43) | 1.05 (0.22) |
| Art Nostalgia | 3.19 (1.37) | 1.31 (0.71) | 3.64 (1.39) | 3.40 (1.33) |
| Art Melancholy | 2.33 (1.22) | 1.36 (0.79) | 2.36 (1.38) | 2.29 (1.40) |
| Art Personal meaning | 4.14 (0.93) | 1.14 (0.52) | 4.69 (0.47) | 4.48 (0.63) |
| Art Beauty | 3.79 (0.90) | 1.10 (0.37) | 4.33 (0.72) | 4.19 (0.77) |
| Art Chills | 1.67 (0.98) | 1.02 (0.15) | 2.33 (1.26) | 2.33 (1.26) |
| Art Enjoyment | 4.36 (0.69) | 1.59 (0.86) | 4.59 (0.63) | 4.64 (0.62) |
| Art Congruency | - | - | - | 4.67 (0.53) |
