# Supplementary material for: Registered Report: How does art impact pain and stress? Exposure to multimodal art (Music + Visual) and music alone enhances pain tolerance more than visual art, but neither art form impacts autonomic or endocrine markers
Source: PLoS One. 2026 May 5;21(5):e0334060. doi: 10.1371/journal.pone.0334060 (PMC13143110; doi:10.1371/journal.pone.0334060)
Supplement: S12 Table — (DOCX) [file pone.0334060.s015.docx]

**S12 Table. ANOVAs of Mechanisms of Pain and Stress as well as Features of the Arts**

| Measures | Effect | df | *F* | *p* | Generalized *η*² | *BF_10_* ± *Margin of Error %* |
| --- | --- | --- | --- | --- | --- | --- |
| Distraction | Condition | 3, 123 | 127.33 | *≤ 0.0001***** | 0.651 | 2.242×10 ^38^  ± 0.67% |
| Mind wandering | Condition | 3, 123 | 88.65 | *≤ 0.0001***** | 0.527 | 9.904×10 ^28^  ± 0.92% |
| Art Pleasantness | Condition | 3, 123 | 163.34 | *≤ 0.0001***** | 0.755 | 2.346×10 ^48^  ± 0.97% |
| Art Emotional Arousal | Condition | 3, 123 | 155.48 | *≤ 0.0001***** | 0.728 | 3.030×10 ^42^  ± 0.94% |
| Art Liking | Condition | 3, 123 | 229.04 | *≤ 0.0001***** | 0.801 | 3.603×10 ^56^  ± 0.7% |
| Art Joy | Condition | 3, 123 | 226.18 | *≤ 0.0001***** | 0.773 | 3.737×10^56^  ± 1.24% |
| Art Sadness | Condition | 3, 123 | 1.65 | 0.182 | 0.023 | 0.239  ± 4.76% |
| Art Relaxation | Condition | 3, 123 | 68.98 | *≤ 0.0001***** | 0.559 | 3.495×10^39^  ± 1.21% |
| Art Anger | Condition | 3, 123 | 1.47 | 0.227 | 0.023 | 0.192  ± 0.64% |
| Art Fear | Condition | 3, 123 | 1.32 | 0.271 | 0.019 | 0.155  ± 0.48% |
| Art Nostalgia | Condition | 3, 123 | 49.03 | *≤ 0.0001***** | 0.36 | 2.209×10^18^  ± 0.44% |
| Art Melancholy | Condition | 3, 123 | 11.16 | *≤ 0.0001***** | 0.108 | 1.140×10^4^  ± 0.53% |
| Art Personal meaning | Condition | 3, 123 | 323.37 | *≤ 0.0001***** | 0.829 | 7.621×10^66^  ±0.58% |
| Art Beauty | Condition | 3, 123 | 295.60 | *≤ 0.0001***** | 0.775 | 7.255×10^60^  ±0.52% |
| Art Chills | Condition | 3, 123 | 28.46 | *≤ 0.0001***** | 0.225 | 1.817×10^11^  ±1.01% |
| Art Enjoyment | Condition | 3, 123 | 204.31 | *≤ 0.0001***** | 0.770 | 1.351×10^52^  ±0.5% |

*Note. All these scales were rated on a 5-point Likert type scales ranging from 1: Not at all, to 5: Very much. Significance levels: *: p ≤ 0.05; **: p ≤ 0.01; ***: p ≤ 0.001; ****: p ≤ 0.0001.*

*Bayes Factor interpretation. BF_10_ provides evidence for two competing hypotheses, and it shows the ratio of the likelihood of the data falls under one hypothesis to the likelihood that the data falls under the other hypothesis. BF_10_ indicates the Bayes factor comparing the alternative hypothesis (H_1_) to the null hypothesis (H_0_). Interpretation: BF₁₀ < 1: Evidence for the null hypothesis (H_0_), BF_10_ between 1 and 3: Anecdotal evidence for the alternative hypothesis (H_1_), BF_10_ between 3 and 10: Moderate evidence for H_1_, BF_10_ between 10 and 30: Strong evidence for H_1_, BF_10_ between 30 and 100: Very strong evidence for H_1_, BF₁₀ > 100: Extreme evidence for H_1_* [9,10]*.*
