# Supplementary material for: Registered Report: How does art impact pain and stress? Exposure to multimodal art (Music + Visual) and music alone enhances pain tolerance more than visual art, but neither art form impacts autonomic or endocrine markers
Source: PLoS One. 2026 May 5;21(5):e0334060. doi: 10.1371/journal.pone.0334060 (PMC13143110; doi:10.1371/journal.pone.0334060)
Supplement: S13 Table — (DOCX) [file pone.0334060.s016.docx]

**S13 Table.** **Qualitative Data Coding Scheme for Pairs of Visual Artworks and Music Pieces**

| **Category** | **Definition** | **Response options** |
| --- | --- | --- |
| **Autobiographical memory** | The person has chosen the artwork or piece of music because it reminds them of a memory from their own life (i.e., an autobiographical memory). They mention a specific autobiographical event, timeframe, person, or location (e.g., childhood, last summer, my father, my home country). | ***Yes:*** Person explicitly mentions a memory from their life.  ***No***: Person does not mention such memory.  ***NA***: Cannot be assessed based on the quote. |
| **Feature of the artwork/music piece** | The person has chosen the artwork or piece of music because of its features—for example, colour, luminance, etc., for visual artworks, and melody for musical pieces. | ***Yes***: Person explicitly mentions such feature of the art.  ***No:*** Person does not mention such features.  ***NA:*** Cannot be assessed based on the quote. |
| **Emotion regulation** | Person has chosen the artwork/piece of music because it makes them feel some emotion  (e.g. mood lifting) | ***Yes:*** People explicitly mention that the art makes them feel a certain way.  ***No:*** Person does not talk about emotions.  ***Just Emotions:*** Person mentions emotions, but it did not necessarily make them feel something  For example, they associate the artwork with these emotions/feel the artwork expresses these emotions.  ***NA:*** Cannot be assessed based on the quote. |
| **Meaning Making** | The person has chosen the artwork or piece of music because it evokes life-related thoughts—such as reflections on the meaning of life, transcendent emotions, or broader meaning-making processes (e.g., the artwork prompts them to reflect on their approach to life, growing older, or similar existential themes). | ***Yes:*** Person explicitly mentions that the art makes them think about larger questions in life, deeper meanings.  ***No:*** Person does not talk about meaning making, deeper thoughts about life.  ***NA:*** Cannot be assessed based on the quote. |
| **Mind wandering** | The person has chosen the artwork or piece of music because it causes their mind to wander to a specific place or triggers the imagination of places they would like to be. | ***Yes:*** Person explicitly mentions the desire to make their mind wander or to be at a specific imagined place.  ***No:*** Person does not explicitly mention mind wandering or a place where they would like to be.  ***NA:*** Cannot be assessed based on the quote. |
