# Supplementary material for: Registered Report: How does art impact pain and stress? Exposure to multimodal art (Music + Visual) and music alone enhances pain tolerance more than visual art, but neither art form impacts autonomic or endocrine markers
Source: PLoS One. 2026 May 5;21(5):e0334060. doi: 10.1371/journal.pone.0334060 (PMC13143110; doi:10.1371/journal.pone.0334060)
Supplement: S14 Table — (DOCX) [file pone.0334060.s017.docx]

**S14 Table. Representative Quotes and Their Coding**

The following table presents exemplary codes and how they were finally coded. While the first two quotes were assigned exclusively to one category, the third quote shows statements from all categories.

| **Quote** | **Autobiographical memory** | **Feature artwork/ music piece** | **Emotion**  **regulation** | **Meaning**  **meaking** | **Mind**  **wandering** |
| --- | --- | --- | --- | --- | --- |
| 1. The song is my favourite and reminds me of summer and my girlfriends.  I associate the picture with my art lessons at school, which I really liked. | Yes | No | No | No | No |
| 2. I find the artwork beautiful because the color composition is very aesthetic & dreamy.  The song goes well with it, as it is also dreamy and imaginative & the melody is easy to remember. | No | Yes | No | No | No |
| 3. Yes, I think the two go well together.  I recently went to see the Monet exhibition and this painting caught my attention. I like it so much because, on the one hand, I would love to be in a place like that right now and, on the other hand, I love the sea more than anything. I find that when I look at the picture, my own problems seem so small. The beauty of nature, the mood and also a special feeling are triggered in me when I look at it.  I find the piece of music very fitting, because it is calm but also very dynamic, like the different states of the sea. That is why I chose this one, because I play the piano myself and whenever I play this piece, I am happy.  Addition: the artwork is by Monet and is called "By the sea". | Yes | Yes | Yes | Yes | Yes |

**References**

1. Melzack R. The McGill Pain Questionnaire: Major properties and scoring methods. Pain. 1975 Sep 1;1(3):277–99.

2. Oesch P, Eberhardt R, Hilfiker R, Keller S, Kool J, Luomajoki H, et al. Assessments in der Rehabilitation - Band 2: Bewegungsapparat. Hogrefe Verlag; 2017.

3. Hautzinger M, Keller F, Kuehner C. Beck Depressions-Inventar Revision (BDI-II) Manual. 2009;

4. Löwe B, Spitzer R, Zipfel S, Herzog W. PHQ-D: Gesundheitsfragebogen für Patienten; Manual Komplettversion und Kurzform. 2002;

5. Ditzen B, Nussbeck F, Drobnjak S, Spörri C, Wüest D, Ehlert U. Validierung eines deutschsprachigen DSM-IV-TR basierten Fragebogens zum prämenstruellen Syndrom. Z Klin Psychol Psychother. 2011 Oct 21;40(3):149–59.

6. Reniers RLEP, Corcoran R, Drake R, Shryane NM, Völlm BA. The QCAE: A questionnaire of cognitive and affective empathy. J Pers Assess. 2011;93(1):84–95.

7. Ritz T, Dahme B. Die Absorption-Skala: Konzeptuelle Aspekte, psychometrische Kennwerte und Dimensionalität einer deutschsprachigen Adaptation. Diagnostica. 1995;41(1):53-61.

8. Tellegen A, Atkinson G. Openness to absorbing and self-altering experiences (“absorption”), a trait related to hypnotic susceptibility. J Abnorm Psychol. 1974 Jun;83(3):268–77.

9. Lee MD, Wagenmakers EJ. Bayesian model comparison. In: Bayesian Cognitive Modeling [Internet]. Cambridge University Press; 2014. p. 101–17. Available from: https://www.cambridge.org/core/product/identifier/CBO9781139087759A049/type/book_part

10. Wagenmakers EJ, Lodewyckx T, Kuriyal H, Grasman R. Bayesian hypothesis testing for psychologists: A tutorial on the Savage–Dickey method. Cogn Psychol [Internet]. 2010 May;60(3):158–89. Available from: https://linkinghub.elsevier.com/retrieve/pii/S0010028509000826

11. Posada-Quintero HF, Florian JP, Orjuela-Cañón ÁD, Chon KH. Highly sensitive index of sympathetic activity based on time-frequency spectral analysis of electrodermal activity. American Journal of Physiology-Regulatory, Integrative and Comparative Physiology [Internet]. 2016 Sep 1;311(3):R582–91. Available from: https://www.physiology.org/doi/10.1152/ajpregu.00180.2016

12. Posada-Quintero HF, Derrick BJ, Winstead-Derlega C, Gonzalez SI, Claire Ellis M, Freiberger JJ, et al. Time-varying Spectral Index of Electrodermal Activity to Predict Central Nervous System Oxygen Toxicity Symptoms in Divers: Preliminary results. In: 2021 43rd Annual International Conference of the IEEE Engineering in Medicine & Biology Society (EMBC) [Internet]. IEEE; 2021. p. 1242–5. Available from: https://ieeexplore.ieee.org/document/9629924/

13. Kong Y, Posada-Quintero HF, Chon KH. Pain Detection using a Smartphone in Real Time. In: 2020 42nd Annual International Conference of the IEEE Engineering in Medicine & Biology Society (EMBC) [Internet]. IEEE; 2020. p. 4526–9. Available from: https://ieeexplore.ieee.org/document/9176077/

14. Kong Y, Posada-Quintero HF, Chon KH. Sensitive Physiological Indices of Pain Based on Differential Characteristics of Electrodermal Activity. IEEE Trans Biomed Eng. 2021;68(10):3122–30.
