# Supplementary material for: Registered Report: How does art impact pain and stress? Exposure to multimodal art (Music + Visual) and music alone enhances pain tolerance more than visual art, but neither art form impacts autonomic or endocrine markers
Source: PLoS One. 2026 May 5;21(5):e0334060. doi: 10.1371/journal.pone.0334060 (PMC13143110; doi:10.1371/journal.pone.0334060)
Supplement: Attachment — Submitted filename: Response to Reviewers.docx [file pone.0334060.s018.docx]

Manuscript number: PONE-D-25-45648

Title: Registered Report: How does Art Impact Pain and Stress? Exposure to Multimodal Art (Music + Visual) and Music Alone Enhances Pain Tolerance More Than Visual Art, but Neither Art Form Impacts Autonomic or Endocrine Markers

***Dear Editor,***

***Thank you for the opportunity of resubmitting our manuscript.***

***Dear Editor, Dear Reviewers,***

***Below we respond first to the comments of the editor, followed by the responses to the reviewers (written in bold and italics). Note all page numbers indicated in the response letter are based on the manuscript without tracked changes.***

***Response to the Editor:***

Journal Requirements:

When submitting your revision, we need you to address these additional requirements.

1. Please ensure that your manuscript meets PLOS ONE's style requirements, including those for file naming. The PLOS ONE style templates can be found at

<https://journals.plos.org/plosone/s/file?id=wjVg/PLOSOne_formatting_sample_main_body.pdf> and

<https://journals.plos.org/plosone/s/file?id=ba62/PLOSOne_formatting_sample_title_authors_affiliations.pdf>

***Thank you very much. We have ensured that our manuscript meets PLOS ONE's style requirements, including those for file naming.***

2. Thank you for stating the following in the Acknowledgments Section of your manuscript:

“This research was funded by two grants to Anna Fekete and to Rosa Maidhof, who both received a grant (“Förderstipendium”) from the University of Vienna, Büro Studienpräses.”

We note that you have provided funding information that is not currently declared in your Funding Statement. However, funding information should not appear in the Acknowledgments section or other areas of your manuscript. We will only publish funding information present in the Funding Statement section of the online submission form.

Please remove any funding-related text from the manuscript and let us know how you would like to update your Funding Statement. Currently, your Funding Statement reads as follows:

“The author(s) received no specific funding for this work.”

Please include your amended statements within your cover letter; we will change the online submission form on your behalf.

***Thank you for your note about the funding. We have addressed this point in the Cover Letter.***

3. We note that the grant information you provided in the ‘Funding Information’ and ‘Financial Disclosure’ sections do not match.

When you resubmit, please ensure that you provide the correct grant numbers for the awards you received for your study in the ‘Funding Information’ section.

***The grants that we had received is an internal university financial support grant and were without an official reference number.***

4. Thank you for stating the following financial disclosure:

 “This research was funded by two grants to Anna Fekete and to Rosa Maidhof, who both received a grant (“Förderstipendium”) from the University of Vienna, Büro Studienpräses.”

At this time, please address the following queries:

a) Please clarify the sources of funding (financial or material support) for your study. List the grants or organizations that supported your study, including funding received from your institution. 
b) State what role the funders took in the study. If the funders had no role in your study, please state: “The funders had no role in study design, data collection and analysis, decision to publish, or preparation of the manuscript.”
c) If any authors received a salary from any of your funders, please state which authors and which funders.
d) If you did not receive any funding for this study, please state: “The authors received no specific funding for this work.”
Please include your amended statements within your cover letter; we will change the online submission form on your behalf.

***Thank you for this comment. We addressed it in the Cover Letter.***

5. Please remove your figures from within your manuscript file, leaving only the individual TIFF/EPS image files, uploaded separately. These will be automatically included in the reviewers’ PDF.

***We removed our figures from within our manuscript files and left the individual image files separately.***

6. If the reviewer comments include a recommendation to cite specific previously published works, please review and evaluate these publications to determine whether they are relevant and should be cited. There is no requirement to cite these works unless the editor has indicated otherwise. 

***Thank you, we reviewed all recommendations to cite specific previously published works and only added them if we found them relevant to our work.***

1. Does the manuscript adhere to the experimental procedures and analyses described in the Registered Report Protocol?

If the manuscript reports any deviations from the planned experimental procedures and analyses, those must be reasonable and adequately justified.

Reviewer #1: Yes

Reviewer #2: Yes

2. If the manuscript reports exploratory analyses or experimental procedures not outlined in the original Registered Report Protocol, are these reasonable, justified and methodologically sound?


A Registered Report may include valid exploratory analyses not previously outlined in the Registered Report Protocol, as long as they are described as such.

Reviewer #1: Yes

Reviewer #2: Partly

3. Are the conclusions supported by the data and do they address the research question presented in the Registered Report Protocol?


The manuscript must describe a technically sound piece of scientific research with data that supports the conclusions. The conclusions must be drawn appropriately based on the research question(s) outlined in the Registered Report Protocol and on the data presented.

Reviewer #1: Yes

Reviewer #2: Partly

4. Have the authors made all data underlying the findings in their manuscript fully available?

The [PLOS Data policy](http://track.editorialmanager.com/CL0/http:%2F%2Fwww.plosone.org%2Fstatic%2Fpolicies.action%23sharing/1/010f019bba4ef1d3-62ba63f6-008e-450e-9ab3-ebfe41e4e5cb-000000/-Fd3p0jPaxJ5-D0ObPTtD-G2QTJ1WTsz6D9CS6z1r6o=245) requires authors to make all data underlying the findings described in their manuscript fully available without restriction, with rare exception (please refer to the Data Availability Statement in the manuscript PDF file). The data should be provided as part of the manuscript or its supporting information, or deposited to a public repository. For example, in addition to summary statistics, the data points behind means, medians and variance measures should be available. If there are restrictions on publicly sharing data—e.g. participant privacy or use of data from a third party—those must be specified.

Reviewer #1: Yes

Reviewer #2: Yes

5. Is the manuscript presented in an intelligible fashion and written in standard English?

*PLOS ONE* does not copyedit accepted manuscripts, so the language in submitted articles must be clear, correct, and unambiguous. Any typographical or grammatical errors should be corrected at revision, so please note any specific errors here.

Reviewer #1: Yes

Reviewer #2: Yes

***Dear Reviewers,***

***Thank you for taking the time to review our manuscript and for your constructive feedback.***

***Response to Reviewer 1:***

Reviewer #1: General Evaluation
The manuscript presents a carefully designed and methodologically rigorous registered report examining how multimodal (music + visual art) aesthetic experiences influence experimentally induced pain and stress. The authors have implemented a well-controlled within-subjects design, preregistered their procedures, and ensured full data transparency via OSF (<https://osf.io/yxgrv/>).
The study makes a valuable contribution to empirical aesthetics and psychophysiology by demonstrating that multimodal and music-only conditions significantly enhance pain tolerance compared with visual art and control, while physiological stress markers remain unchanged. The paper is detailed, intellectually rich, and methodologically transparent.

1. Assessment of the Literature Review
The literature review is generally comprehensive, well-structured, and logically organized around three major research areas: (1) music and pain/stress, (2) visual art and pain/stress, and (3) multimodal or combined art experiences.
Strengths:
The authors accurately synthesize core meta-analyses and seminal studies (e.g., Lee, 2016; de Witte et al., 2020; Mitchell et al., 2008; de Tommaso et al., 2008).
The review effectively links aesthetic engagement with psychological mechanisms of pain modulation (attention, emotion, meaning-making), referencing Howlin & Rooney (2020).
The authors appropriately identify inconsistencies in prior findings on visual art and position their work as addressing these gaps.

***We thank the reviewer for the positive assessment of our manuscript.***

Areas for improvement:
The review could benefit from the inclusion of more recent work in multimodal and neuroaesthetic research, such as Brattico & Vuust (2017) and Koelsch (2020), to reinforce the neural and cognitive basis for cross-modal integration.
The discussion of individual difference variables (trait absorption and empathy) is thoughtful but could be expanded by referencing newer frameworks, such as Aesthetic Responsiveness (Schlotz et al., 2020) or Musical Aesthetic Sensitivity (Clemente & Nadal, 2022), which would contextualize these traits within contemporary personality–aesthetics research.
The introduction may briefly address the growing literature on digital versus in-person art experiences (e.g., Specker et al., 2023), since the present study used digital reproductions of artworks.
The number of citations is extensive and might be streamlined by emphasizing recent meta-analyses to enhance readability.
Overall, the literature review is well-developed and conceptually solid, warranting only minor updates to incorporate the latest findings in multimodal and individual-difference research.

***Thank you for the suggestion. As a Registered Report, our Stage 2 Introduction and Methods intentionally mirror the approved Stage 1 protocol (https://doi.org/10.1371/journal.pone.0266545), following guidance (https://doi.org/10.1371/journal.pcbi.1010571) to limit changes to tense shifts and factual corrections. Our academic editor confirmed this approach: keep these sections largely unchanged, with minor clarifications; substantial shortening is discouraged. For PLOS ONE examples, you may see:*** [***https://journals.plos.org/plosone/search?filterJournals=PLoSONE&filterArticleTypes=Research%20Article&q=registered%20report%20protocol&page=1***](https://journals.plos.org/plosone/search?filterJournals=PLoSONE&filterArticleTypes=Research%20Article&q=registered%20report%20protocol&page=1) ***´.***

***Therefore, we do not have the opportunity to implement the suggested references.***

***Regarding the comment on digital reproductions, we discuss this issue in our Discussion section:***

***“A related issue is that of stimulus quality. In the visual art condition, we used the digital reproductions of the artworks, and not genuine artworks. Similarly, for the music condition, we used a digital recording—not a live concert. Nevertheless, there might be differences between the quality of a professionally recorded music piece and a photo of an artwork on the screen. An important difference here is that recorded music pieces (that we used in the study) can be seen as the ‘original’ work, which cannot be said for the reproductions of artworks we used (see also [150, 151]. Therefore, future studies might consider the effect of genuine artworks (i.e., original paintings in the laboratory) on pain perception, which might provide similar extent of distraction or mind wandering like music listening.” (p.46)***

2. Study Rationale and Theoretical Framing
The rationale is strong and socially relevant. The authors clearly outline the public health importance of nonpharmacological pain management and the potential of aesthetic experiences. However, the theoretical link to the Vienna Integrated Model of Art Perception (VIMAP) could be elaborated further to clarify how the hypothesized mechanisms (distraction, mind wandering, absorption) align with the model’s top–down and bottom–up components.

***Thank you, indeed this could have been a good suggestion for the Stage 1 review, but unfortunately, we cannot make changes in the literature review due to the PLOS ONE guidelines associated with Registered Reports (see also comment 1).***

3. Methods and Procedures
The within-subject design and counterbalancing are exemplary. The authors report methodological details with commendable precision, including apparatus, physiological recordings, and saliva sampling.
Clarification is recommended regarding whether color and luminance calibration were standardized for the visual stimuli, as this can influence perceived beauty and emotional impact.

***We did not standardize color or luminance for the stimulus images. Stimuli were presented using PsychoPy and scaled proportionally to fill the display, but no color or luminance adjustments were applied.***

***For our stimulus set, luminance standardization was not appropriate: enforcing uniform luminance across diverse content (e.g., night scenes vs. snowy landscapes) would produce unrealistic, desaturated images, potentially altering the very aesthetic and emotional qualities under study.***

***The same monitor was used for all participants, so the calibration was consistent. The monitors were not hardware-calibrated. We note that calibration is more critical for paradigms where precise luminance control affects performance (e.g., tasks requiring fast reaction times), rather than studies prioritizing ecological validity of complex, naturalistic stimuli.***

***In summary, we do not think that this was a limitation of the study as we prioritized ecological validity in the present study and therefore, we did not modify the stimuli material.***

The female-only, non-artist sample is justified for experimental control, yet the authors should explicitly acknowledge its limitation for generalizability.

***We agree with the reviewer on this limitation.***

***We discuss this limitation:***

***“In addition, our study may be limited in its generalizability, due to having a female-only sample consisting of only Western, Educated, Industrialized, Rich and Democratic (WEIRD; [156]) participants, and the extensive exclusion criteria we applied (see above) necessary for the accurate measurement of salivary cortisol and alpha-amylase. In general, the more homogenous the sample, the more unclear the extent to which it will generalize. In addition, specifically, it may be that using only females overestimates the found effect, given that females have been found to have higher sensitivity to music [91]. Furthermore, inherently from the nature of laboratory pain studies, there is a sampling bias as to who will volunteer to participate in pain research. Specifically, it is more likely that those people will attend pain studies who have lower level of fear of pain and higher level of sensation-seeking [157]. This could explain why the CPT only partially influenced the saliva stress biomarkers, emphasizing that the effects here should only be interpreted with caution.” (p. 48-49)***

Providing mean ± SD for immersion durations in each condition would enhance interpretability of the pain-tolerance results.

***We would like to note that people immersed their hands in cold water, and this immersion time serves as a measure of pain tolerance (see more details on this measure in the Pain measures section, p.22).***

***We have included the mean and SD of immersion duration = pain tolerance (in s) to Table 1. Descriptive of Pain and Stress Measures: Pain tolerance (in s): Visual M=56.63 (SD=47.86), Control M= 59.50 (SD=55.86), Music M =81.34 (SD=64.19), Multimodal M= 80.19 (SD=61.05) (p.32-33).***

4. Results and Interpretation
The results are internally consistent and presented transparently. The primary findings—greater pain tolerance under music and multimodal conditions—are statistically robust and clearly reported.

***We would like to thank the reviewer for the overall positive evaluation of our results and their interpretation.***

The absence of significant differences in physiological stress indices is plausible and well discussed, but a note regarding statistical power for endocrine measures (given high individual variability) would be helpful.

***Thank you for this comment, we agree and added this limitation to the discussion section (p. 49): “Furthermore, we note the limitation*** ***that our sample size was calculated based on our main behavioural outcome (pain intensity), so physiological and endocrine measures were likely underpowered. Future studies should account for these measures when determining sample size.”***

The exploratory analyses on distraction and mind wandering are valuable and could be strengthened with a simplified conceptual figure illustrating their proposed mediating roles.

***Thank you for the suggestion. Although a conceptual figure could aid interpretation, we did not conduct formal mediation analyses; rather, we ran exploratory regressions. After careful consideration, we decided not to include a mediation figure to avoid implying unsupported causal pathways.***

The qualitative data analysis of participants’ motivations adds richness to the manuscript; including one or two representative quotes in the Supplementary Information would provide readers with a more tangible sense of the responses.

***We agree that including one or two representative quotes would be helpful for a deeper understanding of the responses. We have now added selected exemplary quotes in the Supplementary Information (S14 Table, p. 18). While the first two quotes were assigned exclusively to one category (1.: Autobiographical memory; 2.: Feature artwork/ music piece), the third quote shows statements from all possible categories. The selected quotes were the following (with more details in the Supplementary Information):***

***1. The song is my favourite and reminds me of summer and my girlfriends. I associate the picture with my art lessons at school, which I really liked.***

***2. I find the artwork beautiful because the color composition is very aesthetic & dreamy. The song goes well with it, as it is also dreamy and imaginative & the melody is easy to remember.***

***3. Yes, I think the two go well together. I recently went to see the Monet exhibition and this painting caught my attention. I like it so much because, on the one hand, I would love to be in a place like that right now and, on the other hand, I love the sea more than anything. I find that when I look at the picture, my own problems seem so small. The beauty of nature, the mood and also a special feeling are triggered in me when I look at it. I find the piece of music very fitting, because it is calm but also very dynamic, like the different states of the sea. That is why I chose this one, because I play the piano myself and whenever I play this piece, I am happy. Addition: the artwork is by Monet and is called "By the sea".***

5. Discussion and Implications
The Discussion is comprehensive and integrates findings across multiple domains. However, the first three paragraphs (pp. 46–48) contain overlapping statements regarding Hypotheses 1–2 and could be condensed for readability.

***We thank the reviewer for this helpful suggestion. We have condensed the first three paragraphs to make them more concise and readable.***

The authors’ interpretation of trait absorption as a significant moderator is novel and insightful. The null finding for empathy could be further contextualized by distinguishing between cognitive and affective empathy and their differing relevance for aesthetic engagement.

***We are grateful for this valuable comment. We have further elaborated on this idea in the text (p. 43):***

***Fourth, we found no effect of trait empathy on pain or stress across conditions, contrasting with prior work suggesting greater music-induced pain relief among individuals with high trait empathy [80]. However, higher cognitive empathy was associated with lower pain unpleasantness, independent of condition. Whereas affective empathy involves sharing others’ feelings, cognitive empathy involves understanding them [138]. One possibility is that individuals higher in cognitive empathy focused on understanding the CPT experience and thereby cognitively regulated it rather than feeling it. In line with this, Thompson et al. [138] suggested that cognitive empathy relates to better emotion regulation, whereas affective empathy relates to greater emotion regulation difficulties.***

The discussion of music’s temporal dynamics as an explanatory factor is compelling; linking this to theoretical frameworks such as temporal entrainment or predictive coding would enrich the argument.

***We would like to thank the reviewer for the suggestion, which we have implemented in the discussion section (p.46):***

***Our findings align with two broader frameworks in music research and beyond. First, temporal entrainment, synchronization among independent rhythmic processes [150], may underlie effects unique to music, which has inherent rhythms that can couple with our bodily rhythms, unlike stationary visual art. Second, predictive coding suggests that the brain generates models of the world refined by sensory input, a form of informed “guessing” [151]. Because music is structured by rules that invite predictions and let the listeners anticipate how it will unfold, whereas visual art offers a more static, holistic impression, predictive coding may play a larger role in music than in visual art.***

In conclusion, the authors may wish to expand on the applied implications, highlighting how art-based interventions could be adapted for clinical pain management or mental health contexts.

***We agree this section needed more detail; we have now expanded it (p.46):***

***“In clinical settings, using the attention-engaging, emotionally charged music may strengthen pain-reducing effects. Importantly, personality traits and gender must be considered.*** ***Although emotionally significant, self-selected favourite music was linked to the highest pain tolerance, women who strongly focus on music’s emotional aspects (“music empathizers”) were highly sensitive to pain when listening to their favourites [147]. Therefore, follow-up studies are urgently needed before clinical recommendations can be made.”***

6. Figures, Tables, and Presentation
Figures 3–7 are clear and well designed. The authors should ensure all legends include n values and that statistical annotations remain consistent throughout.

***We revised the figures by adding N values to the legends and ensuring consistency in the statistical annotations.***

Minor typographical and stylistic issues (e.g., inconsistent spacing in Table 2, repeated use of “aesthetic experience”) should be corrected during revision.

***We have revised the figures, tables, and presentation regarding typographical and stylistic issues. In Table 2, we have made spacing consistent. In the parts that we were able to change (please note again, that the Stage 1 Registered Report Protocol of the Introduction and Method section cannot be changed), we ensured a reader-friendly use of “aesthetic experience”.***

Overall, the manuscript is clearly written and requires only light language editing.

***We appreciate the overall positive assessment of our manuscript and have performed language editing to improve the paper.***

Summary Recommendation
The manuscript is technically sound, conceptually coherent, and methodologically transparent. The literature review is comprehensive but would benefit from modest updates to reflect recent multimodal and individual-difference research.

***We thank Reviewer 1 for the constructive feedback and evaluation; we believe the comments improved the manuscript.***

***Response to Reviewer 2:***

Reviewer #2: Major Comments

1. Abstract: lack of quantitative results

The Abstract does not report any quantitative information (e.g., means, effect sizes, confidence intervals) and therefore does not adequately convey the empirical strength of the findings. Given that most outcomes showed no differences across conditions, a more quantitative and transparent abstract is particularly important for PLOS One readers. The authors should revise the Abstract to clearly report key effect sizes and null results.

***We thank the reviewer for this helpful comment. We have revised the abstract and added more quantitative information to convey the empirical strength of the findings. As part of the revision of the abstract, we have adjusted its length to stay within the specified word limit.***

2. Length and structure of the manuscript

The manuscript is considerably longer than necessary relative to the core empirical contribution. The Introduction and theoretical sections are highly detailed and at times repetitive, particularly in the discussion of mechanisms and multimodal aesthetics. Substantial shortening is recommended, especially by reducing theoretical elaboration that is not directly tested or supported by the reported results. Improving focus would greatly enhance readability and impact.

***Thank you for this observation. Due to the Registered Report format, we have copied the Introduction and Methods section from our Stage 1 protocol paper (***[***https://doi.org/10.1371/journal.pone.0266545***](https://doi.org/10.1371/journal.pone.0266545)***). We followed the practice suggested by the literature (e.g.*** [***https://doi.org/10.1371/journal.pcbi.1010571***](https://doi.org/10.1371/journal.pcbi.1010571)***) that suggests that “Your introduction and method sections in your Stage 2 paper should not deviate unnecessarily from your approved Stage 1 manuscript, other than changing from future to past tense (e.g., “we will test” changes to “we tested”) or correcting any factual errors or misunderstandings." (Rule 9: Prepare your Stage 2 manuscript)***

***We have contracted the academic editor on this who confirmed this approach “It is recommended to keep the Stage 1 Introduction and Methods largely unchanged in the Stage 2 manuscript. The main edits should be changing tenses from future to past, correcting anything that was overlooked, and making clarifications. Significant shortening is generally discouraged but small adjustments for clarity and readability are ok provided the Stage 1 content remains without abrupt changes.”***

***We refer to further examples in PlosOne with Registered Report Stage 1 protocol papers and Registered Report Article paper (stage 2 paper):*** [***https://journals.plos.org/plosone/search?filterJournals=PLoSONE&filterArticleTypes=Research%20Article&q=registered%20report%20protocol&page=1***](https://journals.plos.org/plosone/search?filterJournals=PLoSONE&filterArticleTypes=Research%20Article&q=registered%20report%20protocol&page=1)

3. Clarity regarding outcomes and analysis strategy

Although the Methods section is very detailed, it is not always clear which outcomes constitute primary confirmatory endpoints versus secondary or exploratory analyses. Given the Registered Report format, this distinction is crucial. The authors should more explicitly identify primary outcomes and hypotheses in the main text and ensure that the Results and Discussion are structured accordingly.

***We agree that clearly distinguishing primary confirmatory endpoints from secondary or exploratory analyses is essential.***

***We have articulated our confirmatory hypotheses: “In the present study, we—for the first time—systematically tested: (1) the effect of multimodal (music, visual, and combined) aesthetic experience on pain. (2) stress-responsive systems; and in both cases, compared the single modalities (e.g., testing whether music or visual art is more effective); in an exploratory manner, (3) investigated the underlying mechanisms of aesthetic experience; and (4) examined the individual differences.” (Present study section, p. 15, followed by the Hypotheses section.)***

***Accordingly, we have organized our Results section in line with this principle: first we report the manipulation checks (p.29) and in line with the hypotheses we labelled the confirmatory analyses with the heading*** “***Confirmatory Analyses: Effects of Aesthetic Experience on Pain” (p.32), Confirmatory Analyses: Effects of Aesthetic Experience on Stress (p.34) and the exploratory analyses with the following heading: “Exploratory Analyses: Mechanisms of Aesthetic Experience on Pain and Stress”(p.36).***

***Due to the guidelines of PLOS ONE regarding Registered Reports, we cannot change the Introduction and Methods sections (see comment 1 and comment 1 of the first reviewer).***

***We hope that these changes have made it clear what type of analyses are presented in each section.***

4. Interpretation of null findings and discussion scope

The study reports largely null effects for autonomic and endocrine stress markers. While these findings are valid and important, the Discussion sometimes extrapolates mechanistic interpretations that are not clearly supported by the physiological data. The authors should adopt a more restrained interpretation and ensure that conclusions closely track the empirical evidence, particularly regarding proposed underlying mechanisms.

***We thank the reviewer for this valuable comment. We revised the discussion to limit claims to those supported by physiological data and to frame other points as tentative. Conclusions now align more closely with the evidence, especially regarding mechanisms.***

5. Generalizability and framing of implications

The study sample is highly specific (young, healthy female participants with strict exclusion criteria). While this is methodologically justified, the implications for broader pain and stress management should be framed more cautiously. Claims regarding potential applications or general benefits of art exposure should be explicitly limited to the studied population and context.

***We agree with the reviewer on this limitation.***

***We discuss this limitation:***

***“In addition, our study may be limited in its generalizability, due to having a female-only sample consisting of only Western, Educated, Industrialized, Rich and Democratic (WEIRD; [156] participants, and the extensive exclusion criteria we applied (see above) necessary for the accurate measurement of salivary cortisol and alpha-amylase. In general, the more homogenous the sample, the more unclear the extent to which it will generalize. In addition, specifically, it may be that using only females overestimates the found effect, given that females have been found to have higher sensitivity to music [91]. Furthermore, inherently from the nature of laboratory pain studies, there is a sampling bias as to who will volunteer to participate in pain research. Specifically, it is more likely that those people will attend pain studies who have lower level of fear of pain and higher level of sensation-seeking [157]. This could explain why the CPT only partially influenced the saliva stress biomarkers, emphasizing that the effects here should only be interpreted with caution.” (p. 50)***

***We have revised the discussion with regard to its generalizability and the framing of the implications. In doing so, we have formulated the implications more cautiously and placed them in the context of the specific sample.***

I have not provided minor, line-by-line comments, as the key issues concern structure, focus, and interpretation rather than surface-level edits.

***We thank Reviewer 2 for the constructive feedback and evaluation; we believe the comments improved the manuscript.***
