# Supplementary material for: Registered Report: How does art impact pain and stress? Exposure to multimodal art (Music + Visual) and music alone enhances pain tolerance more than visual art, but neither art form impacts autonomic or endocrine markers
Source: PLoS One. 2026 May 5;21(5):e0334060. doi: 10.1371/journal.pone.0334060 (PMC13143110; doi:10.1371/journal.pone.0334060)
Supplement: Attachment — Submitted filename: Response_to_Reviewers_auresp_2.docx [file pone.0334060.s019.docx]

PONE-D-25-45648R1

Registered Report: How does Art Impact Pain and Stress?

Exposure to Multimodal Art (Music + Visual) and Music Alone Enhances Pain Tolerance More Than Visual Art, but Neither Art Form Impacts Autonomic or Endocrine Markers

***Dear Editor,***

***Thank you for the opportunity of resubmitting our manuscript.***

***Dear Reviewers,***

***Thank you for taking the time to review our manuscript again and for your constructive feedback.***

***Below we respond to each of the points raised (written in bold and italics). Please note all page numbers indicated in the response letter are based on the manuscript without tracked changes.***

***Response to Reviewer 1:***

Reviewer #1: Reviewer Comments to the Authors

General Evaluation

I would like to thank the authors for their thorough and thoughtful response to my previous comments. The revised manuscript has been significantly strengthened, and the authors have demonstrated a commendable level of scientific rigor in addressing each of the concerns raised. This Stage 2 Registered Report now provides a highly transparent and well-structured presentation of the findings regarding the impact of art on pain and stress. I am particularly satisfied with how the authors managed the balance between the inherent constraints of the Registered Report format and the need for greater empirical clarity.

Specific Feedback on Revisions

1. Quantitative Transparency in the Abstract The inclusion of specific means, standard deviations, and p-values in the Abstract is a major improvement. It now allows the reader to immediately grasp the empirical strength of the findings, particularly the notable enhancement of pain tolerance in the music and multimodal conditions. This level of detail is essential for a high-quality PLOS ONE publication.

2. Clarity of the Analytic Strategy I appreciate the reorganization of the Results section. The explicit use of headings for "Confirmatory Analyses" and "Exploratory Analyses" effectively guides the reader through the study’s logic and clearly distinguishes between pre-planned hypothesis testing and subsequent hypothesis-generating investigations. This approach greatly enhances the transparency of the work.

3. Justification for Manuscript Structure and Length I accept the authors' justification regarding the Stage 2 manuscript guidelines. Given that the Introduction and Methods must remain consistent with the approved Stage 1 protocol, the authors have done an excellent job of making minor adjustments to enhance readability without violating the Registered Report requirements.

4. Interpretation of Null Findings and Generalizability The revisions to the Discussion are well-noted. By acknowledging that the physiological and endocrine measures may have been underpowered, the authors provide a more scientifically grounded interpretation of the null results. Furthermore, the more cautious framing of the clinical implications within the context of the specific study sample (WEIRD, female-only) adds to the credibility of the conclusions.

***Thank you for the positive evaluation of our revision, we are glad to hear that you found the revised manuscript clearer.***

A Minor Suggestion for the Final Version

Regarding the significant main effects reported in Table 2 for "Pain intensity" (p = .038) and "Pain affect" (p = .025), the authors correctly state that the post-hoc pairwise comparisons were not significant after Bonferroni correction. To ensure absolute clarity for future readers, I recommend that the authors conduct a final brief audit of the phrasing in these specific result paragraphs to ensure there is no ambiguity between the existence of an omnibus effect and the lack of specific group-level differences.
I am pleased to recommend this manuscript for publication and look forward to its contribution to the field of empirical aesthetics.

***Thank you for this comment. We have now made a change:***

***“For pain intensity and pain affect (unpleasantness), there was a significant effect of condition, but the effect diminished in the pairwise post hoc comparisons. Thus, although the omnibus tests indicate that at least one condition may differ, the corrected post hoc tests did not identify specific group-level differences (Table 1, Table 2, Fig 4B, Fig 4C).” (p. 33)***

***Response to Reviewer 2:***

Reviewer #2: Thank you for the careful revision. The manuscript has improved, and the revised version is clearer in several important respects. In particular, the distinction between confirmatory and exploratory analyses is now more explicit, the abstract is more informative and quantitative, and the limitations regarding generalizability are better acknowledged.

***Thank you for the positive evaluation of our revision, we are glad to hear that you found the revised version much improved.***

I do have some comments that can be improved for the final version:
1. Exploratory analyses
The exploratory analyses are now more clearly identified and appear broadly reasonable. However, their justification and methodological strength are not fully convincing in all cases, particularly with regard to mechanistic interpretations.

2. Mechanistic interpretation
Some of the broader interpretive claims, especially those related to underlying mechanisms, still seem more tentative than the confirmatory results warrant. I encourage the authors to keep these interpretations cautious and clearly framed as exploratory.

***We have now rephased the interpretations of the mechanisms with a more cautious language, using “may”, “might” or “seems” instead of “do” or “is”. Similarly, we wrote “was associated with” instead of more predictive claims. In addition, we have clearly stated that these analyses were conducted in an exploratory way.***

***You can find the rewritten parts in the Discussion section:***

***“To connect these findings of the mechanisms of art experience (distraction, mind wandering) and the role of trait absorption, we tested in an exploratory way whether people higher in absorption would be also more distracted from the pain and have more mind wandering, but we found no evidence for this assumption. However, we did find that trait absorption was associated with mind wandering in the visual only condition. Though only explorative, this analysis suggests that the strength of the manipulation (see also below) might be less relevant for people high in absorption, due to their higher proneness to mind wandering (thus reaching similar levels regardless of condition). Given that mind wandering is predictive of pain tolerance, the findings suggest that people high in absorption may be likely better at tolerating pain (at least when exposed to aesthetic stimuli).” (p.44)***

***And***

***“Taken together, these findings suggest that the experience itself seems to be important, that is, art seems to be an effective way to increase pain tolerance, potentially because it may distract from the pain and make people’s mind wander away from the pain.” (p.45)***

***And***

***“These considerations are based on our exploratory analyses and are in line with findings on aesthetic experience and self-referential processes activating the default mode network [101, 145, 146].” (p.46)***

3. Conclusions and scope of inference
The main conclusions regarding pain tolerance and the absence of consistent effects on stress markers are generally supported by the data and do address the core research questions. However, the broader implications should remain aligned with the strength of the findings.

***We thank the reviewer for this comment. To better align our conclusions and the scope of inference with the strength of our findings, we have significantly weakened the broader implications in the discussion section in the following way:***

- ***“One possible explanation for our findings might be that individuals higher in cognitive empathy may have focused on understanding the CPT experience and thereby may have cognitively regulated it rather than feeling it.” (p.43-44)***
- ***In line with comment 2: “To connect these findings of the mechanisms of art experience (distraction, mind wandering) and the role of trait absorption, we tested in an exploratory way whether people higher in absorption would be also more distracted from the pain and have more mind wandering, but we found no evidence for this assumption. However, we did find that trait absorption was associated with mind wandering in the visual only condition. Though only explorative, this analysis suggests that the strength of the manipulation (see also below) might be less relevant for people high in absorption, due to their higher proneness to mind wandering (thus reaching similar levels regardless of condition). Given that mind wandering is predictive of pain tolerance, the findings suggest that people high in absorption may be likely better at tolerating pain (at least when exposed to aesthetic stimuli).” (p.44)***
- ***In line with comment 2: “Taken together, these findings suggest that the experience itself seems to be important, that is, art seems to be an effective way to increase pain tolerance, potentially because it may distract from the pain and make people’s mind wander away from the pain.” (p.45)***
- ***In addition: “It seems that the music might create a more attention-engaging and emotionally loaded experience which may improve the effect of the visual art in pain and stress experiences.” (p.45-46)***
- ***In line with comment 2: “These considerations are based on our exploratory analyses and are in line with findings on aesthetic experience and self-referential processes activating the default mode network [101, 145, 146].” (p.46)***
- ***“In addition, regarding the null effects for our other pain measures, it is possible that attending the pain exposure on different days might have influenced our results.” (p.48)***
- ***“In consideration of our results, it seems possible that there might be a dose-relationship here, with the stressor having to be of moderate intensity for music—or art in general—to reduce stress and potentially pain.” (p.49)***

***In the abstract, we have emphasized that the underlying mechanisms need further research. “In conclusion, exposure to art can influence pain; however, the underlying mechanisms require further research.” (Abstract)***

***Furthermore, we also added more specific recommendations for future research regarding the mechanisms in order to make clear that our findings do not allow clinical recommendations:***

***“Therefore, follow-up studies about how these mechanisms of the experience and the person work are urgently needed before clinical recommendations can be made.” (p.46)***

4. Final framing
In the final version, it would be helpful to maintain a clear distinction throughout between supported confirmatory findings and more speculative or hypothesis-generating interpretations.

***Thank you for these comments. We have now further improved the distinctions between the confirmatory and exploratory analyses and their interpretations further.***

***For example, we added to the discussion section whether the hypotheses were confirmatory or exploratory:***

***“Our results partly supported these confirmatory hypotheses, both multimodal art and music enabled people tolerate pain longer than single modality of visual art.” (p.42)***

***“We did not find evidence for this confirmatory hypothesis as the art modalities did not differ in their stress influencing capacities.” (p.43)***

***“Fourth, regarding the exploratory analyses of underlying mechanisms of the person, we found no effect of trait empathy on pain or stress across conditions, contrasting with prior work suggesting greater music-induced pain relief among individuals with high trait empathy [80].” (p.43)***

***In addition, we framed explicitly that the mechanisms of the person (individual differences) were also exploratory:***

***“Fourth, regarding the exploratory analyses of underlying mechanisms of the person, we found no effect of trait empathy on pain or stress across conditions, contrasting with prior work suggesting greater music-induced pain relief among individuals with high trait empathy [80].” (p.43)***

***In line with comments 2 and 3: “To connect these findings of the mechanisms of art experience (distraction, mind wandering) and the role of trait absorption, we tested in an exploratory way whether people higher in absorption would be also more distracted from the pain and have more mind wandering, but we found no evidence for this assumption.” (p.44)***

***In line with comments 2 and 3: “These considerations are based on our exploratory analyses and are in line with findings on aesthetic experience and self-referential processes activating the default mode network [101, 145, 146].” (p.46)***

***We hope we have now differentiated clearly which interpretations are based on the confirmatory findings and which ones are based on the exploratory ones.***
